# Longitudinal, mixed method study to look at the experiences and knowledge of non melanoma skin cancer from diagnosis to one year

**DOI:** 10.1186/1471-5945-13-13

**Published:** 2013-10-29

**Authors:** Fiona Bath-Hextall, Claire Jenkinson, Arun Kumar, Jo Leonardi-Bee, William Perkins, Karen Cox, Cris Glazebrook

**Affiliations:** 1 School of Health Sciences, Faculty of Medicine & Health Sciences, University of Nottingham, Queen’s Medical Centre, Nottingham NG7 2UH, UK; 2 Division of Psychiatry, School of Community Health Sciences, Queen’s Medical Centre, Nottingham NG7 2UH, UK; 3 Division of Epidemiology and Public Health, Clinical Sciences Building, City Hospital, Nottingham NG5 1PB, UK; 4 Department of Dermatology, Queen’s Medical Centre, University Hospital, Nottingham NG7 2UH, UK; 5 Nottingham Clinical Trials Unit, University of Nottingham, Queen’s Medical Centre, Nottingham NG7 2UH, UK

**Keywords:** Skin cancer, Non melanoma skin cancer, NMSC, Needs, Experiences, Knowledge

## Abstract

**Background:**

Skin cancer is the most common type of cancer in humans and the incidence is increasing worldwide. Our objective was to understanding the needs, experiences and knowledge of individuals with Non Melanoma Skin Cancer (NMSC) from diagnosis up until one year.

**Methods:**

Patients with NMSC completed questionnaires at diagnosis, treatment, 8 weeks post treatment and 12 months post diagnosis. Body image, psychological morbidity and Quality of Life (QOL) were assessed at each time point, with the exception of QOL that was not assessed at diagnosis. Knowledge of NMSC was assessed at baseline and 8 weeks. A sub-sample of participants was also interviewed to allow a more in-depth exploration of patients’ experiences.

**Results:**

76 participants completed the initial questionnaire, of which 15 were interviewed. Patients were anxious about a diagnosis of skin cancer, however they were no more depressed or anxious than the general population. QOL significantly improved from diagnosis to 8 weeks and from diagnosis to one year. Knowledge of NMSC was poor and did not improve after treatment. Hairdressers were highlighted as playing an important role in raising awareness and encouraging individuals to seek medical help. Most participants were aware of the need to check their skin for suspicious lesions but were not sure what to look for. At one year participants had forgotten their experience and were not overly concerned about skin cancer.

**Conclusion:**

There is a need to raise awareness of the signs and symptoms of NMSC. Information on skin cancer needs to be tailored to the individual both at the start of treatment and during the follow up months, ensuring that participants’ needs and expectations are met. Targeting education at individuals in the community who regularly come into contact with skin should help in early identification of NMSC. This is important since skin cancer caught early is easily treatable and delay in presentation leads to larger and more complex lesions which impacts in terms of increased morbidity and increased health care costs.

## Background

Skin cancer is the most common type of cancer in humans [1], accounting for 20% of all cancers diagnosed in the UK. Around 97% of skin cancers are epithelial in origin and are either basal cell carcinomas (BCCs) or squamous cell carcinomas (SCCs), collectively known as non-melanoma skin cancer (NMSC). A systematic review of the worldwide incidence of Non Melanoma Skin Cancer (NMSC) [2] has shown that the incidence of NMSC varies widely with highest rates in Australia (> 1000/100,000 person-years for BCC) and lowest rates in parts of Africa (< 1/100,000 person-years for BCC). The average incidence rates in England were 76.21/100,000 persons and 22.65/100,000 persons for BCC and SCC respectively. The incidence rates in the UK appear to be increasing at a greater rate when compared to the rest of Europe. NMSC is most common in older age groups but the incidence of BCC is increasing in the young [3-6]. There are many options for the treatment of NMSC and these have been reviewed elsewhere [7-9].

The National Institute for Clinical Excellence (NICE) guidelines for improving care of people with skin tumours [10] highlighted a lack of research into the needs and experiences of people with skin cancer and the need for high quality patient information.

A systematic review looking at qualitative research alone [11] found only two papers that examined the needs and experiences of people with skin cancer (melanoma and/or non-melanoma skin cancer). The studies identified by the review focused mainly on melanoma skin cancer and did not investigate patients’ perceptions of their information, support, and decision making needs over the course of their illness, or the extent to which these had been met. A study in 40 patients exploring patients’ understanding of pigmented skin lesions and skin cancer concluded that changing moles were often perceived as trivial and not signifying possible skin cancer [12]. A recently published study looked at 982 consecutive American patients presenting for Mohs micrographic surgery for NMSC and found that 71% of people delayed going to their doctor with NMSC because they were in denial about the severity of their condition [13]. It is known that early diagnosis can dramatically improve prognosis and the patient experience since early lesions are treated more simply compared with larger or neglected lesions [13]. It has been shown that patients with melanoma, BCC and SCC experience similar levels of anxiety and depression after diagnosis and treatment [14], and surgery for skin cancer has been shown to impact on their appearance [15]. Health professionals working with skin cancer patients need to understand the psychosocial concerns of this patient group in order to design services appropriately and to provide patients with the support they need and information that they can easily understand. The patient experience is an important component in the evidence based cycle. Research in this area has predominantly focused on malignant melanoma. This study combines both quantitative and qualitative methods to follow skin cancer patients on their journey from diagnosis to one year post diagnosis.

## Methods

A mixed method study, using postal questionnaires and semi structured interviews. Our objective was to understand the needs, experiences and knowledge of individuals with Non Melanoma Skin Cancer (NMSC) from diagnosis up until one year.

Consecutive patients, referred from primary care, attending a skin cancer clinic in a large teaching hospital in the East Midlands over an 8 month period in 2008/9 with a new clinical diagnosis of NMSC, were invited to take part in the study. No recurrent cases were included however patients with previous skin cancer were not excluded. Ethical approval was obtained from the Nottingham Research Ethics Committee 1 (08/H0403/83) and signed consent was obtained from all participants.

**Figure 1:**
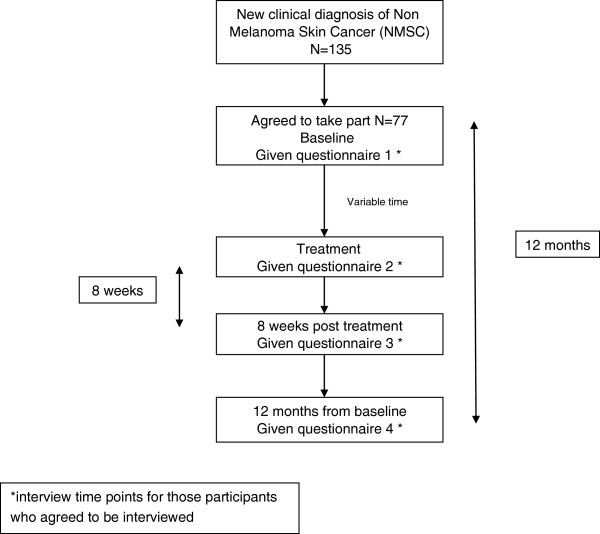
Study flow diagram.

Questionnaires (Additional files 1 and 2) were sent to patients at four time points: baseline (just after diagnosis, usually the next day), treatment (same day or next day), 8 weeks post-treatment and 12 months from baseline. Body image and psychological morbidity were assessed at each time point using the *Derriford Appearance Scale 24 (DAS24)*[16] and the *Hospital Anxiety and Depression Scale (HADS)*[17]*.* Knowledge of NMSC was assessed at baseline and 8 weeks post-treatment. This instrument was adapted from the melanoma knowledge questionnaire [18] in consultation with dermatologists and piloted prior to use. Demographics and sun exposure data were collected at baseline and participants’ concerns about how NMSC affected their quality of life were assessed after treatment, 8 weeks post-treatment and at 12 months from baseline using the *Skin Cancer Index (SCI)*[19]*.* See study flow chart, Figure 1. SCI was not assessed at baseline as the questions were deemed inappropriate for patients who had just received a diagnosis of NMSC.

We invited all participants to take part in an interview. Purposive sampling ensured we captured a proportion of younger people (< 60 yrs). Interviews were designed to elicit in-depth views, perceptions and descriptions of the experience of being treated for NMSC, recovery from treatment and feelings 12 months after diagnosis. A flexible interview guide (Additional file 3) was used to ensure consistency across the interviews while allowing interviewees to express their ideas, understanding and concerns freely.

Anxiety and Depression were measured using the Hospital Anxiety and Depression Scale (HADS), a 14-item self-rating scale, with questions pertaining to the past week, in medical outpatients, primary care and community settings, which have been validated for cancer patients. The HADS was answered by the participant on a four-point (0–3) response category with a possible score of 0 to 21 for anxiety (7 items) and for depression (7 items). For each construct, a score below 8 was classified as 'normal’, 8–10 as 'borderline’ and above 10 as 'caseness’ (probable presence of disorder).

The Derriford Appearance Scale (DAS24) is a 24-item scale designed to measure distress and dysfunction in relation to problems of appearance. Some of the items request a response about the intensity of emotional response, using response categories of 'extremely’ to 'not at all’ (e.g. 'How distressed do you get when you see yourself in the mirror/window?’). Other items ask about the frequency of particular behaviours indicative of a self-conscious response (e.g. 'I avoid going out of the house’), using an 'almost always’ to 'never/almost never’ set of response categories. Where appropriate, a response of 'not applicable’ is allowed. There is no threshold score or cut-off point to indicate 'caseness’. The minimum score is 11 and the maximum is 96 (highest distress/dysfunction).

Knowledge of NMSC was ascertained using three free-response questions to assess knowledge about how to reduce risk from NMSC (one mark for each of avoiding sunburn, not using sun beds, checking skin frequently), what type of people are at risk of NMSC (one mark for each of fair skin, sunbather, work outdoors, use of sun beds, previous skin cancer/family history of skin cancer, older age), and early skin signs of NMSC (one mark for stating any of the following: lump/pearly lump/change in lump/sore/unusual area/new area/recently grown plus one mark for mentioning a surface change that is non-healing :crusting, scabbing, bleeding plus half a mark for using the word raised). The maximum possible score is 11.5.

*The Skin Cancer Index (SCI)* is a 15 item, validated, disease-specific QOL instrument with 3 distinct subscales, Emotion, Social, and Appearance. Higher scores reflect better quality of life [20]. Summary statistics were computed for each SCI question, the three SCI subscale totals, and the overall SCI total. The subscales were transformed so that each subscale, and the total, was between 0 and 100.

All quantitative analyses was performed using SPSS version 16.0. Summary statistics were computed for the HADS anxiety and depression, DAS24, Knowledge of NMSC, and total SCI. 95% confidence intervals for the means of each outcome were calculated at various time points and for the change over time windows. We used paired t test for examining the HADS, DAS24 and also the SCI index. We calculated the mean change in HADS anxiety and depression and DAS24 across three different time windows: baseline – 12 months post baseline, treatment – eight weeks post treatment, and treatment – 12 months post baseline. We also calculated the mean change in SCI across 2 different time windows: treatment - 8 weeks post treatment and treatment - 12 months post baseline.

Interviews were transcribed and analysed using thematic content analysis [21]. The qualitative data analysis package NVivo8 was used to deductively and inductively derive codes and check that these harmonized with the context of the interview statement. Codes were grouped into clusters and categorised into major themes. Members of the team collectively analysed and discussed the emerging themes.

## Results

One hundred and thirty five patients with an initial clinical diagnosis of NMSC were referred to the researcher by the clinician of whom 77 (57%) agreed to take part in the study and returned a consent form. The clinical diagnosis was confirmed in all but one person who was then excluded. The mean age of the group was 70 years (range 35–89), 40 (53%) were male and a quarter reported having had a previous skin cancer. BCC was the most common (80%), the majority of which were on the head or neck, Table 1. Twenty nine percent of the participants had outdoor occupations and 20% of participants had lived abroad. Over half of the participants had sunburn (causing pain/discomfort/peeling) as a child (32% had 1–2 episodes and 25% had > = 3 episodes). Seventeen percent had sunburn in the last two years (38% were under 60 years of age and 12% were over 60 years of age). Seventy seven percent of participants spent between 1–4 weeks on holiday and 11% spent between 5–12 weeks on holiday. Seven percent of participants aged > 60 yrs frequently sunbathed compared to 14% in the 40–60 year olds and 33% of those < 40 yrs. Of the participants who sunbathed, 78% used sunscreen although most participants were unsure as to the SPF.

**Table 1:** Demographic characteristic of participants

**Characteristics**	**No (%) of participants**
Age, yrs	
30-49	5 (7)
50-59	11 (15)
60-69	15 (20)
70-79	28 (37)
80-89	17 (22)
Sex	
Male	40 (53)
Female	36 (47)
Marital status	
Single	5 (7)
Married	47 (62)
Living with partner	5 (7)
Widowed	14 (8)
Divorced	5 (7)
Previous skin cancer	
No	56 (74)
Yes	19 (25)
Missing	1 (1)
Histology	
BCC	61 (80)
SCC	14 (18)
Missing	1 (1)
Site	
Head/neck	60 (79)
Rest of body	15 (20)
Missing	1 (1)
Diameter (mm)	
1-4	12 (16)
5-9	36 (47)
> = 10	19 (25)
Missing	9 (12)
HADS	Median (IQR) 6.0 (1–11)
Anxiety	
0-7	63 (84)
8-10	8 (11)
> = 11	4 (5)
	Median (IQR) 3.0 (1–6)
Depression	
0-7	70 (93)
8-10	2 (3)
> = 11	3 (4)
	Median (IQR) 2.0 (0–5)
DAS-24 (N = 69)	Mean (SD) 23.7 (8.8)

SD standard deviation.

BCC Basal Cell Carcinoma.

SCC Squamous Cell Carcinoma.

The time from first clinic visit to treatment varied from 0 to 159 days (median 41 days, IQR 23.5 - 57.5 days). Sixty-eight participants were treated by excision, one was treated by excision followed by radiotherapy and 8 were treated by Mohs micrographic surgery. The mean time from diagnosis to treatment for Mohs micrographic surgery was 101 days (SD 33.33, range 47–159 days). The mean time from diagnosis to treatment for excision was 37.18 days. Two patients received diagnosis and treatment on the same day and resultant histology confirmed their clinical diagnosis. Participants’ demographics and disease characteristics are shown in Table 1.

The mean age of the interviewees was 66 (range 39–88). Four were aged ≤ 60 years. Thirteen were clinically diagnosed with BCC and 2 with SCC. Six participants (40%) were male and 3 (20%) reported having had a previous skin cancer. The site of skin cancer was located on the head for all but one interviewee whose lesion was on the torso. Ten of the participants sunbathed regularly, five had used sun beds, four had been sunburnt in the past 2 years, five spent more than 50% of their holidays abroad, three had lived abroad in hot countries and two remembered getting sunburnt as a child.

### Quantitative results

Baseline, treatment, 8 weeks post-treatment and 12 months post-baseline questionnaires were returned by 76, 67, 67, and 66 participants respectively. A number of participants did not return the questionnaires at various time points but no reasons were given. A number of participants withdrew (one due to broken ankle, three due to a broken limb, one lesion was not a NMSC, one gave no reason and one died (not related to NMSC) 8 weeks post treatment.

Baseline scores for HADS anxiety and depression were classified as normal for 84% (anxiety) and 93% (depression) of the participants respectively. The DAS24 scores were consistent with those reported by a general (non-patient) population (Table 1). Knowledge of NMSC was low (range 0–7.5) with a mean total score of 3.28/11.5 (SD 1.61). There were no significant gender differences with respect to anxiety (p = 0.087), depression (p = 0.274), DAS24 (p = 0.213), or knowledge (p = 0.371). Knowledge was no better in participants who had previously had a skin cancer. A significant positive correlation was found between age and depression with older people having higher depression scores (Rs = 0.374, p = 0.001). A significant positive correlation was seen between the HADS Anxiety score and DAS24 score (Rs = 0.454, p < 0.001), with those with higher DAS24 scores (poorer body image) having a higher anxiety score.

Quality of life significantly improved from treatment to 8 weeks post treatment (p = 0.034) and from treatment to 12 months post baseline (p = 0.001) (Table 2). There was no significant change in body image from baseline to 12 months post baseline or from treatment to 8 weeks post treatment (Table 2). There was no significant change in knowledge of NMSC from baseline to 8 weeks post treatment (Table 3).

**Table 2:** Changes in quantitative outcomes over time

**Scale**	**Comparison Mean (95% CI)**	**P value**
DAS-24	Baseline to 12 months post diagnosis (N = 59)	0.289
	23.9 (21.5,26.3) to 23.0 (20.9,25.2)	
	Treatment to 8 week post treatment (N = 61)	0.306
	23.0 (20.8,25.3) to 22.2 (20.2,24.2)	
HADS		
Anxiety	Baseline to 12 months post diagnosis (N = 62)	0.003
	3.6 (2.7,4.4) to 2.5 (1.8,3.3)	
	Treatment to 8 week post treatment (N = 59)	0.026
	3.1 (2.2,3.9) to 2.5 (1.7,3.2)	
Depression	Baseline to 12 months post diagnosis (N = 59)	0.003
	2.9 (2.0,3.7) to 1.9 (1.2,2.6)	
	Treatment to 8 week post treatment (N = 59)	0.848
	2.2 (1.5,2.9) to 2.2 (1.4,2.9)	
SCI	Treatment to 8 weeks post treatment (N = 63)	0.034
	78.21 (73.8, 82.6) to 82.3 (78.6, 85.9)	
	Treatment to 12 months post diagnosis (N = 57)	0.001
	78.4 (73.8, 83.0) to 85.5 (82.0, 89.0)	

**Table 3:** Knowledge of Non Melanoma Skin Cancer (NMSC)

	**Baseline**	**8 weeks after treatment**	Mean difference (95% CI)*	**P value for difference**
Knowledge Category	n, Mean (SD)	n, Mean (SD)		
Reduce Risk	76, 1.00 (0.40)	67, 1.02 (0.41)	0.03 (-0.07-0.13)	0.57
Risk Type	76, 1.39 (0.97)	67, 1.30 (0.80)	-0.10 (-0.36-0.15)	0.42
Signs of NMSC	76, 0.89 (0.78)	67, 0.72 (0.70)	-0.19 (-0.41-0.04)	0.10
Total Knowledge	76, 3.28 (1.61)	67, 3.03 (1.28)	-0.32 (-0.69-0.05)	0.09

*Difference in scores based on 67 participants with baseline and 8 weeks after treatment scores.

Although mean anxiety and depression scores were in the normal range (≤ 8.0) at each time point (Table 2), we found that patients became even less anxious (p = 0.003) and depressed (p = 0.003) 12 months post diagnosis (Table 2).

### Qualitative results

Forty one participants (53%) consented to be interviewed. Of these 15 were interviewed. These 15 were selected to give us a broad a range of views and experiences as possible covering gender, age (above and below 60). The interviews were conducted over the telephone with the exception of one interviewee who was interviewed face to face. The interviews were audio-taped and fully transcribed. There were no significant differences in demographics between the participants interviewed and those comprising the whole study.

*At baseline (just after diagnosis)* four major themes emerged from analysis of the 15 interviewees : *Chance and cue to action*; *Rationalisation for delay in seeking medical help*; *Reaction to clinical diagnosis* and *I’m quite aware now*. The themes and their variations are described below and highlighted by comments from interviewees.

#### Chance and cue to action

Participants were asked what prompted them to go to their doctor. Reasons included the inability of their skin condition to heal completely and the persistent cycle of bleeding, scab formation and re-emergence. Others recalled visiting their general practitioner for some other reason and just mentioning in passing a skin condition that they were not particularly concerned about. A number of participants said that their hairdresser (13%) had drawn their attention to the skin lesion and suggested they went to see their general practitioner. Other individuals who encouraged participants to seek advice included a community skin cancer specialist (7%), a dentist (7%) and a friend (7%). A number of participants (some of whom had a previous experience of skin cancer), visited their general practitioner without prompting (66%).

“My hairdresser said to me, a few weeks ago, “The next time you go to the doctor’s mention the mark on your temple,” he said, “because I don’t like the looks of it”… and it wasn’t until I went to the doctor’s about three weeks ago when I thought, "Oh”, I said, “can you just have a look at this mark on my temple, the hairdresser told me to mention it to you.” And he looked at it, and then he got a magnifying glass and he said, “I don’t think it's anything to worry about but I’ll refer you to a dermatologist…”

F, 74, BCC

#### Rationalisation for delay in seeking medical help

Most participants found it difficult to remember exactly when they first noticed the lesion. A lack of awareness that it could be skin cancer was the main reason for delay. The majority were aware of the skin lesion and that it sometimes caused physical discomfort such as irritation or bleeding and failed to heal. The lesion was often attributed to being a minor skin condition, a symptom of old age that might heal on its own and nothing to worry about.

“I’d got a spot that was reoccurring and didn’t know whether it was just old age or whether it was something that was untoward… I didn’t really see it as a priority. I wish I’d have gone earlier but at the time didn’t think it was anything.”

F, 39, BCC

“It’s over five years because I'd had had this tiny red patch…I mean I'm saying five years, it could have been ten years… I just assumed it was definitely a little patch of eczema, so it never occurred to me that it could be skin cancer.”

F, 67, BCC

#### Reaction to clinical diagnosis

The mention of the word 'cancer’ caused alarm and concern for most participants. Concern was mainly about how long they had had the cancer and if it could have spread and if they had other undetected skin cancers. Appearance and potential disfigurement following treatment was another concern. All patients felt they had a good rapport with the dermatologist, however some participants felt their information needs had not been met and thought they should have received more information especially when some patients compared the information given to them with that given to patients with other types of cancer.

“You know, to the person who has actually got it it’s not relatively minor… it’s that big C word again, and while it’s there it can spread. But that’s a personal feeling of mine and I don't know how many people are diagnosed with this type of thing yearly but I bet most people feel the same.”

M, 55, SCC

'I'm quite aware now’

Several participants felt that having experienced a NMSC they would generally know what to look for in future and would have the confidence to act more quickly in presenting to a general practitioner. Others were aware of the need to check the skin but were not sure what to look for. Some participants remained unaware that they needed to check their skin but some participants changed their behaviour.

“*Well, I mean, yeah, if anything, I got a scab and I were picking it well I’d be straight to the doctors*”

F, 69, BCC

“I wear cream all the time. I wear a hat all the time now as well. I don’t sit in the sun, I used to years ago, I used to sit in the sun a lot, but I don’t do that anymore.”

F, 70, BCC

*Immediately post treatment* the themes to emerge were: *Treatment satisfaction; Appearance and information needs; Recurrence and new lesions; Increased awareness.*

#### Treatment satisfaction

All patients were satisfied with their care. For most, treatment satisfaction was associated with the knowledge that their skin cancer had been completely removed.

“The good thing is the bad stuff’s gone and I’m not that vain, if you know what I mean.”

M, 45, BCC

#### Appearance and information needs

Potential disfigurement and scarring was a concern for many patients. Participants were concerned that they had no information about what was happening after the treatments.

“I was a little anxious because I didn’t quite know what I was going to look like when I was finished.”

F, 62, BCC

“Maybe a little bit more information about what the scar would feel like and look like and, you know, I’m not sure whether this lumpiness and the hardness of the scar is going to soften and go away or whether it’s permanent”.

F,67, BCC

#### Recurrence and new lesions

Patients were concerned that although the lesion had been removed, it might come back and they worried about further lumps and bumps.

“Well, it’s just that, you know, I just hope that it’s all gone and that it’s not going to flare up again. I mean, I'm touching wood, I don’t think it will do because I don’t think it was that sort of thing, but there's bound to be doubts, small doubts, you know, even though people do try and reassure you. But hopefully everything will be okay.”

F, 74, BCC

#### Increased awareness

Patients were more aware of skin cancer and were taking steps to avoid getting another lesion. They were checking for new lesions, although some were not sure what they were looking for. Participants also said they were spreading the word to others about the need to check their skin.

“… it has raised awareness significantly in our family now, yeah....I've been into town today and bought a moisturiser with SPF25 or something or other, so yeah. So I'll wear that constantly now rather than general moisturisers.”

F, 39, BCC

“I am [checking], yes, but I’m not too sure what to look for.... Now if I see anything that’s not, I’ll be, instead of waiting I shall be straight to the doctor’s and see what it is.”

M, 45, BCC

*8 weeks post treatment* the main themes to emerge were: *Information needs about checking skin; Spreading the word.*

Patients understood the need to continue to check for further skin lesions although most were not sure what they were looking for. Many participants continued to spread the word about skin cancer advising others to seek medical advice regarding suspicious lesions.

#### Information needs about checking skin

“Well I mean in my experience the problem has not known what to look for. I mean if all these things were identical in appearance, then I would feel a lot easier. But by and large they’ve taken different forms, and it is a question of knowing what to look for ....”

M, 78, BCC

#### Speading the word

“One or two people I have spoken to and I’ve said, I did say look out for dubious looking spots and things that won’t go away with ointments and things.”

F, 79, BCC

*12 months post baseline* the main themes to emerge at one year post diagnosis were *Satisfaction* and *Not sure what to look for?*

One year on, most people were satisfied with the treatment and care they had received. They were happy that their NMSC had been completely removed and that any scaring had healed. Some participants continue to check their skin but were still not sure what to look for. Interestingly, when they spoke about skin cancer they spoke about moles despite having had a non melanoma skin cancer.

#### Satisfaction

“I’m fine, absolutely fine, I don’t really think about it at all. I had this spot and they took it off and its fine. It doesn’t worry me at all.”

F, 64, BCC

Not sure what to look for?

“I wasn’t really given any advice on checking it at all. You know, I was just told that it was sun damage on my face and on my nose but I wasn’t given any advice on how to check it. I mean I know, I've read up a lot about it, so I know that I check and then if there’s things I can’t see like my back, I get my husband to check, and I always check his regularly as well.”

F, 70, BCC

## Discussion

Although not a life threatening disease, a diagnosis of NMSC caused concern for many participants, and whilst this was not evident from the total quantitative data this was evident from those we interviewed. At diagnosis our participants were neither clinically anxious nor depressed; however, levels of anxiety were higher than levels of depression and 16% of our participants experienced significant levels of psychological distress. These results are similar to another study that found 19% of NMSC patient experienced significant levels of psychological distress [22]. Participants who were most anxious had a poorer body image although body image scores were not high and were representative of the general population who are not concerned about their appearance [16]. A review of psychological distress in patients with melanoma also found participants more anxious than depressed [23]. Our study participants were anxious at diagnosis when they heard the word cancer. They were concerned about how long they had had the cancer, whether it had spread and possible disfigurement. Eight weeks after treatment participants were significantly less anxious and when interviewed, said that the concerns they had at treatment no longer existed. By 12 months, participants had forgotten any concerns that they may have had. Anxiety, depression and quality of life all significantly improved post diagnosis.

Knowledge of NMSC was very poor, even among those who had skin cancer previously.

A telephone survey in recently treated Squamous Cell Carcinoma patients in Italy also found skin cancer knowledge to be low [24]. We found no significant difference in knowledge from diagnosis to 8 weeks post treatment. Participants told us that information given at diagnosis was not really considered as they were so shocked at hearing the word 'cancer’ that everything went out of their head and they couldn’t think of anything else. Lack of knowledge of NMSC most likely contributed to the delay in presentation for many of the participants with some not consulting a doctor for up to 2–5 years. Our study found lack of awareness to be one of the main reasons for delay. Despite participants being aware of their skin lesion for many years, most thought it was a sign of ageing or something that would go away despite in many cases the lesion repeatedly bleeding and failing to heal and despite self-treating with various creams. A recent study [13] in patients presenting for Mohs micrographic surgery for NMSC found similar reasons for delay in presentation, however they did not look at what prompted people to eventually seek advice. We found many participants only consulted a doctor after others such as hairdressers, dentist, or friends had encouraged them to do so. Knowledge is a necessary prerequisite for anyone to be able to interpret a symptom as a signal of cancer or to judge it as serious and requiring medical attention [25].

Hairdressers were highlighted in this study as playing an important role in raising awareness and encouraging their clients to seek medical help. A recent study that surveyed hair professionals provides evidence that hair professionals would be receptive to skin cancer education especially as they are already looking for suspicious lesions on customers’ scalp, neck, and face and are acting as lay skin cancer educators [26]. The regular contact hairdressers have with their clients ensures familiarity with their clients’ skin and therefore they are in a good position to see unusual changes on the head and neck. These findings add to the evidence that educating people about skin cancer is crucial in identifying and thus appropriately treating the disease [27]. Two recently published commentaries have considered the possibility of utilizing hairdressers for early detection of head and neck melanoma [28] and have suggested that hair salon operators should perform opportunistic surveillance of their clients to recognise suspicious lesions and then advise that they see their doctor [29].

The main concern of our patients at 12 months after diagnosis was that they were still not sure if they would recognise skin changes that might be skin cancer. It is interesting to note that whilst most of our participants at 12 months were still aware of the need to be cautious in the sun, they talked about checking skin for moles with no mention of Non Melanoma Skin Cancer. Knowledge of the risk factors for skin cancer has been found to increase skin surveillance, which in turn is associated with thinner melanoma tumours [30].

Despite there being no change in body image over the course of the study, participants continued to be concerned about their appearance at 8 weeks post treatment.

Quality of life of our participants improved significantly over time and like those in the SCI validation study by Rhee et al., [19] were skewed to the upper range. Our 8 week post-treatment mean total SCI of 82.2 is 6% higher than the 16 week post-surgery score (77.3) in Rhee’s study of 183 patients with a biopsy proven NMSC of the face or neck referred to a dermatological Mohs micrographic surgery clinic. Our group was older, had a higher percentage of men and relatively few patients underwent Mohs micrographic surgery, factors which may account for our relatively high quality of life scores.

### Strengths and limitations

A strength of our study was that we were able to follow participants over time from diagnosis through treatment and a year following diagnosis. A limitation of this study may be that we recruited from only one out-patient clinic; patients are, however, referred to this large teaching hospital from a very wide area and are representative of the general population. Another limitation may be that we adapted the knowledge questionnaire from a melanoma knowledge questionnaire, although upmost care was taken in doing this in close consultation with clinicians.

## Conclusions

Information on skin cancer needs to be tailored to the individual both at the start of treatment and during the follow up months, ensuring that the participants’ needs and expectations are met. There is a real need to raise awareness of the signs and symptoms of NMSC especially in elderly populations. Knowledge about recognising the signs of skin cancer is particularly important since 44% of patients will develop additional lesions within 3 years [31]. Targeting education at individuals in the community who regularly come into contact with skin should help in early identification of NMSC. This is important since skin cancers caught early are easily treatable and delay in presentation leads to larger and more complex lesions which in turn may increase patient morbidity and increased health care costs.

## Abbreviations

NMSC: Non melanoma skin cancer; QOL: Quality of life; BCC: Basal cell carcinoma; SCC: Squamous cell carcinoma; DAS24: Derriford appearance scale 24; HADS: Hospital anxiety and depression scale; SCI: Skin cancer index.

## Competing interest

All authors declare that they have no competing interest.

## Authors’ contributions

FB-H conceived the study, participated in its design and coordination, was involved in the acquisition of data, analysis, drafting of the initial paper and critically revised the manuscript for important intellectual content. WP contributed to the design of the study, acquisition of data and revised the manuscript critically for important intellectual and clinical content. CJ participated in acquisition of data, analysis, and initial draft of paper and critically revised the manuscript for important intellectual content. AK participated in acquisition of data, analysis and critically revised the manuscript for important intellectual content. JL-B participated in the quantitative data analysis and critically revised the manuscript for important intellectual content. KC participated in the design of the study, the qualitative analysis and revised the manuscript critically for important intellectual content. All authors approved the final version to be published.

## Pre-publication history

The pre-publication history for this paper can be accessed here:

http://www.biomedcentral.com/1471-5945/13/13/prepub

## Supplementary Material

Additional file 1 Questionnaire at baseline. Click here for file

Additional file 2 Questionnaire at treatment. Click here for file

Additional file 3 Interview schedule. Click here for file
