# Supplementary material for: Longitudinal, mixed method study to look at the experiences and knowledge of non melanoma skin cancer from diagnosis to one year
Source: BMC Dermatol. 2013 Oct 29;13:13. doi: 10.1186/1471-5945-13-13 (PMC3819707; doi:10.1186/1471-5945-13-13)
Supplement: Additional file 2 — Questionnaire at treatment. [file 1471-5945-13-13-S2.doc]

### Interview Topic Guides

***
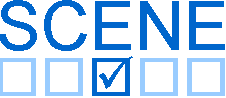
***

**Skin Cancer: Exploring Needs and Experiences**

***Each interview will begin and end with prepared statements***

**Opening statement:**

Thank you for agreeing to be interviewed. I would just like to check again – are you happy for us to audio-tape this interview? We have a series of areas we would like to explore with you rather than a list of questions so I'll start with the first topic if that is OK.

**Closing statement:**

Thank you again for your time. Is there anything else you feel we haven't covered in the interview or anything you would like to clarify? Is there anything else you would like to ask about this study?

**Initial interview - an exploration of care experiences to date, understanding of diagnosis/implications, and initial information/support needs:**

- What was your reason for seeking medical help and did anyone prompt you?
- Did you delay in seeking medical advice and if so why?
- Do you understand what is involved in the management of your skin cancer?
- Do you feel that you have been given adequate support and advice (in particular, advice regarding future surveillance)?
- Do you feel confident/competent to monitor yourself for recurrences or new lesions?

**Follow up interviews to explore:**

Changes over time from initial referral in relation to the above - consider how things may have changed and why

Reflections on the current situation

Social impact – relationships with family/friends

What support is required?

What information is required?
