# Supplementary material for: Longitudinal, mixed method study to look at the experiences and knowledge of non melanoma skin cancer from diagnosis to one year
Source: BMC Dermatol. 2013 Oct 29;13:13. doi: 10.1186/1471-5945-13-13 (PMC3819707; doi:10.1186/1471-5945-13-13)
Supplement: Additional file 3 — Interview schedule. [file 1471-5945-13-13-S3.doc]

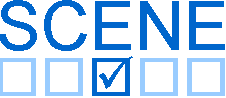


|  |  |  | |  |  |  |  |
| --- | --- | --- | --- | --- | --- | --- | --- |
|  | PID: |  | |  |  |  |  |
|  |  |  | |  |  |  |  |
|  | Questionnaire | | | 1 | of | 4 |  |
|  |  |  | |  |  |  |  |
|  | Received on: | | |  |  |  |  |
|  |  | | |  |  |  |  |
|  | Stage: | | Baseline | | | |  |
|  |  | | |  |  |  |  |

**Skin Cancer: Exploring Needs and Experiences [SCENE]**

Patient questionnaire pack

**Introduction**

In order to help improve our care for patients with a diagnosis like yours we are asking you to inform us about your current thoughts/feelings and recent experiences. We would be grateful if you could complete this questionnaire (following the instructions throughout and answering as appropriate). The questionnaire asks about various topics, including how you feel about your appearance at present and your emotional condition over the last few weeks. Responses to this questionnaire will provide us with an insight into the patient perspective that we do not yet have; enabling us to make recommendations to improve future services.

Please do not feel obliged to respond to this questionnaire. If you do decide to respond, please do not feel obliged to answer every section/question.

If you have any questions about the questionnaire pack, please do not hesitate to approach the researcher who provided you with this questionnaire or contact:

xxxxxxxxxxxxxxxxxxxxxxxxxxxxxxxxxx

xxxxxxxxxxxxxxxxxxxxxxxxxxxxxxxxxxxxxxx

xxxxxxxxxxxxxxxxxxxxxxxxxxxxxxxxxxxxxxxxxxxx

Or write to us at: xxxxxxxxxxxxxxxxxxxxxxxxxxxxxxxxxx


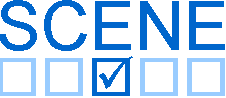


*This part of the questionnaire is concerned with how you feel about your appearance.*

*The first questions are designed to find out if you are sensitive or self-conscious about any aspect of your appearance (even if this is not usually visible to others).*

(a) Is there any aspect of your appearance (however small) that concerns you at all? *(Please tick one box below)*

| Yes | 🞎 |
| --- | --- |
| No | 🞎 |

**If No,** please go to the next page

**If Yes,** please continue

(b) The aspect of my appearance about which I am most sensitive or self-conscious is

…………………………………………………………………………………………………………………………………………………

**From now on, we will refer to this aspect of your appearance as your ‘feature’**

(c) The thing I don’t like about my feature

is …………………………………………………………………………………………………………………………………………….

(d) If you are sensitive or concerned about any other features of your body or your

appearance, please say what they are

…………………………………………………………………………………………………………………………………………………

…………………………………………………………………………………………………………………………………………………

*Please go to the next page*

**
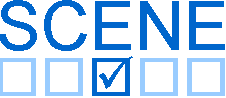
**

*These questions follow on from those on the previous page and are concerned with the way you feel or act. They are all quite simple. For each item, please tick the box under the answer that applies to you. If the item does not apply to you at all, tick the N/A (not applicable) option. Please don’t spend long on any one question.*

|  | Not at all | Slightly | Moderately | Extremely |  |
| --- | --- | --- | --- | --- | --- |
| **How confident do you feel?** | 🞎 | 🞎 | 🞎 | 🞎 |  |
|  | Extremely | Moderately | A Little | Not at all distressed |  |
| **How distressed do you get when you see yourself in the mirror/window?** | 🞎 | 🞎 | 🞎 | 🞎 |  |
|  | N/A | Never/  Almost never | Sometimes | Often | Almost always |
| **My self-consciousness makes me irritable at home:** | 🞎 | 🞎 | 🞎 | 🞎 | 🞎 |
|  | Extremely | Moderately | Slightly | Not at all |  |
| **How hurt do you feel?** | 🞎 | 🞎 | 🞎 | 🞎 |  |
|  | Almost always | Often | Sometimes | Never/  Almost never | N/A |
| **At present my self-consciousness has an adverse effect on my work:** | 🞎 | 🞎 | 🞎 | 🞎 | 🞎 |
|  | N/A | Not at all | A little | Moderately | Extremely |
| **How distressed do you get when you go to the beach?** | 🞎 | 🞎 | 🞎 | 🞎 | 🞎 |
|  | Almost always | Often | Sometimes | Never/  Almost never | N/A |
| **Other people misjudge me because of my feature:** | 🞎 | 🞎 | 🞎 | 🞎 | 🞎 |
|  | Not at all | Slightly | Moderately | Extremely |  |
| **How feminine/masculine do you feel?** | 🞎 | 🞎 | 🞎 | 🞎 |  |


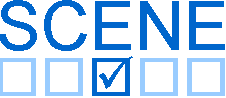


|  | N/A | Never/  Almost never | Sometimes | Often | Almost always |
| --- | --- | --- | --- | --- | --- |
| **I am self-conscious of my feature:** | 🞎 | 🞎 | 🞎 | 🞎 | 🞎 |
|  | Not at all | Slightly | Moderately | Extremely |  |
| **How irritable do you feel?** | 🞎 | 🞎 | 🞎 | 🞎 |  |
|  | Never/  Almost never | Sometimes | Often | Almost always |  |
| **I adopt certain gestures**  (e.g., folding arms in front of other people, covering mouth with hand) | 🞎 | 🞎 | 🞎 | 🞎 |  |
|  | Almost always | Often | Sometimes | Never/  Almost never | N/A |
| **I avoid communal changing rooms:** | 🞎 | 🞎 | 🞎 | 🞎 | 🞎 |
|  | N/A | Not at all | Slightly | Moderately | Extremely |
| **How distressed do you get by shopping in department stores/supermarkets?** | 🞎 | 🞎 | 🞎 | 🞎 | 🞎 |
|  | Not at all | Slightly | Moderately | Extremely |  |
| **How rejected do you feel?** | 🞎 | 🞎 | 🞎 | 🞎 |  |
|  | N/A | Never/  Almost never | Sometimes | Often | Almost always |
| **I avoid undressing in front of my partner:** | 🞎 | 🞎 | 🞎 | 🞎 | 🞎 |
|  | Extremely | Moderately | Slightly | Not at all | N/A |
| **How distressed do you get playing sports/games?** | 🞎 | 🞎 | 🞎 | 🞎 | 🞎 |
|  | Almost always | Often | Sometimes | Never/  Almost never |  |
| **I close into my shell:** | 🞎 | 🞎 | 🞎 | 🞎 |  |


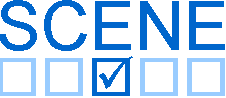


|  | Extremely | Moderately | Slightly | Not at all | N/A |
| --- | --- | --- | --- | --- | --- |
| **How distressed are you by being unable to wear your favourite clothes?** | 🞎 | 🞎 | 🞎 | 🞎 | 🞎 |
|  | N/A | Not at all | Slightly | Moderately | Extremely |
| **How distressed do you get when going to social events?** | 🞎 | 🞎 | 🞎 | 🞎 | 🞎 |
|  | Not at all | Slightly | Moderately | Extremely |  |
| **How normal do you feel?** | 🞎 | 🞎 | 🞎 | 🞎 |  |
|  | Almost always | Often | Sometimes | Never/  Almost never | N/A |
| **At present my self-consciousness has an adverse effect on my sex life:** | 🞎 | 🞎 | 🞎 | 🞎 | 🞎 |
|  | Almost always | Often | Sometimes | Never/  Almost never |  |
| **I avoid going out of the house:** | 🞎 | 🞎 | 🞎 | 🞎 |  |
|  | N/A | Not at all | Moderately | A fair amount | Extremely |
| **How distressed do you get when other people make remarks about your feature?** | 🞎 | 🞎 | 🞎 | 🞎 | 🞎 |
|  | Almost always | Often | Sometimes | Never/  Almost never | N/A |
| **I avoid going to pubs/restaurants:** | 🞎 | 🞎 | 🞎 | 🞎 | 🞎 |
|  | N/A | Never/  Almost never | Sometimes | Often | Almost always |
| **My feature causes me physical pain/discomfort:** | 🞎 | 🞎 | 🞎 | 🞎 | 🞎 |
|  | Almost always | Often | Sometimes | Never/  Almost never |  |
| **My feature limits my physical ability to do the things I want to do:** | 🞎 | 🞎 | 🞎 | 🞎 |  |


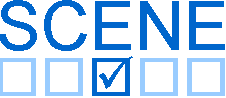


*These questions are designed to let us know how you feel*

*Please read each item and tick the box under the reply that comes closest to how you've been feeling in the past week.*

*Please don't take too long over your replies: your immediate reaction will probably be the most accurate.*

|  | most of the time | a lot of the time | occasionally | not at all |
| --- | --- | --- | --- | --- |
| **I feel tense or wound up** | 🞎 | 🞎 | 🞎 | 🞎 |
|  | definitely as much | not quite as much | only a little | hardly at all |
| **I still enjoy the things I used to enjoy** | 🞎 | 🞎 | 🞎 | 🞎 |
|  | quite badly | not too badly | a little | not at all |
| **I get a sort of frightened feeling as if something awful is about to happen** | 🞎 | 🞎 | 🞎 | 🞎 |
|  | as much as I always could | not quite so much now | definitely not so much now | not at all |
| **I can laugh and as see the funny side of things** | 🞎 | 🞎 | 🞎 | 🞎 |
|  | a great deal of the time | a lot of the time | from time to time | only occasionally |
| **Worrying thoughts go through my mind** | 🞎 | 🞎 | 🞎 | 🞎 |
|  | not at all | not often | sometimes | a lot |
| **I feel cheerful** | 🞎 | 🞎 | 🞎 | 🞎 |
|  | definitely | usually | not often | not at all |
| **I can sit at ease and feel relaxed** | 🞎 | 🞎 | 🞎 | 🞎 |


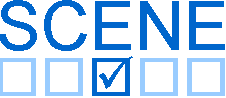


|  | nearly all the time | very often | sometimes | not at all |
| --- | --- | --- | --- | --- |
| **I feel as if I am slowed down** | 🞎 | 🞎 | 🞎 | 🞎 |
|  | not at all | occasionally | quite often | very often |
| **I get a sort of frightened feeling like butterflies in the stomach** | 🞎 | 🞎 | 🞎 | 🞎 |
|  | definitely | I don't take so much care as I should | I may not take quite as much care | I take just as much care as ever |
| **I have lost interest in my appearance** | 🞎 | 🞎 | 🞎 | 🞎 |
|  | very much | quite a lot | not very much | not at all |
| **I feel restless as if I have to be on the move** | 🞎 | 🞎 | 🞎 | 🞎 |
|  | as much as ever | rather less than I used to | definitely less than before | hardly at all |
| **I look forward with enjoyment to things** | 🞎 | 🞎 | 🞎 | 🞎 |
|  | very often | quite often | not often | not at all |
| **I get sudden feelings of panic** | 🞎 | 🞎 | 🞎 | 🞎 |
|  | often | sometimes | not often | very seldom |
| **I can enjoy a good book or programme** | 🞎 | 🞎 | 🞎 | 🞎 |


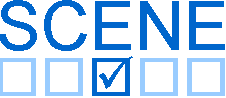


*We are interested in what you understand about non melanoma skin cancer and its treatment.*

*This is a survey,* ***not*** *a test: your answers will help us to identify where information provision could be improved for future patients.*

*Please complete these questions by yourself; your answers will remain anonymous.*

|  | **1. What are the best ways to reduce your risk from non-melanoma skin cancer?** | | | | | |  |
| --- | --- | --- | --- | --- | --- | --- | --- |
|  | (Please write in the box below) | |  |  |  |  |  |
|  |  |  |  |  |  |  |  |
|  |  |  |  |  |  |  |  |
|  |  |  |  |  |  |  |  |
|  | **2. What type of people are more at risk of developing non-melanoma skin cancer?** | | | | | |  |
|  | (Please write in the box below) | |  |  |  |  |  |
|  |  |  |  |  |  |  |  |
|  |  |  |  |  |  |  |  |
|  |  |  |  |  |  |  |  |
|  | **3. What are the signs of non-melanoma skin cancer that you should look out for on your skin?** | | | | | |  |
|  | (Please write in the box below) | |  |  |  |  |  |
|  |  |  |  |  |  |  |  |
|  |  |  |  |  |  |  |  |
|  |  |  |  |  |  |  |  |
|  | **4. What treatments are usually used for non-melanoma skin cancer?** | | | | | |  |
|  | (Please write in the box below) | |  |  |  |  |  |
|  |  |  |  |  |  |  |  |
|  |  |  |  |  |  |  |  |
|  |  |  |  |  |  |  |  |
|  |  |  |  |  |  |  |  |


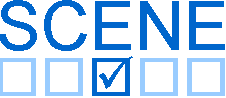


*We would now like to ask you a few questions about sun exposure*

| **What is/was your occupation type?** | |  |  | | | | |  |
| --- | --- | --- | --- | --- | --- | --- | --- | --- |
| Indoor | 🞎 |  | Number of years | | |  | |  |
| Outdoor | 🞎 |  | Number of years | | |  | |  |
| Details of outdoor occupation(s): |  |  |  | | | | |  |
| **How many episodes of sunburn did you have as a child?** | | | | |  | | |  |
| *Include sunburn causing pain/discomfort/peeling etc prior to age 18* | | | | | | | |  |
|  |  |  |  | | | | |  |
| **Do you or have you ever lived abroad?** | |  |  | | | | |  |
| Yes | 🞎 |  |  | | | | |  |
| No | 🞎 |  |  | | | | |  |
| **If yes, how long?** |  | *years* |  | | | | |  |
| **Where?** |  |  |  | | | | |  |
|  |  |  |  | | | | |  |
| **Since age 60,** |  |  |  | | | | |  |
| **on average how many hours during daylight do you spend outdoors per day on:** | | | | | | | | |
| Weekdays |  |  |  | | | | |  |
| Weekends |  |  |  | | | | |  |
| Not applicable (age <60yrs) | 🞎 |  |  | | | | |  |
|  |  |  |  | | | | |  |
| **During your middle age (40-59):** |  |  |  | | | | |  |
| Weekdays |  |  |  | | | | |  |
| Weekends |  |  |  | | | | |  |
| Not applicable (age <40yrs) | 🞎 |  |  | | | | |  |
|  |  |  |  | | | | |  |
| **During early adult life (20-39):** |  |  |  | | | | |  |
| Weekdays |  |  |  | | | | |  |
| Weekends |  |  |  | | | | |  |
| Not applicable (age <20yrs) | 🞎 |  |  | | | | |  |
|  |  |  |  | | | | |  |
| **How many weeks a year do you spend on holiday?** | | | |  | | | |  |
|  |  |  |  | | | | |  |
| **Proportions (lifetime) of holidays spent:** | | | In UK | | | |  | *%* |
|  |  |  | Abroad hot climate | | | |  | *%* |
|  |  |  | Abroad cold climate | | | |  | *%* |


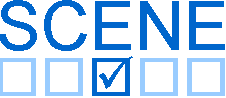


| **Extent of sunbathing/lying/sitting in the sun at following ages?** | | | | |
| --- | --- | --- | --- | --- |
| **60+ years:** |  |  |  |  |
| Frequently | 🞎 |  |  |  |
| Occasionally | 🞎 |  |  |  |
| Rarely | 🞎 |  |  |  |
| Never | 🞎 |  |  |  |
| **40-60 years:** |  |  |  |  |
| Frequently | 🞎 |  |  |  |
| Occasionally | 🞎 |  |  |  |
| Rarely | 🞎 |  |  |  |
| Never | 🞎 |  |  |  |
| **0-40 years:** |  |  |  |  |
| Frequently | 🞎 |  |  |  |
| Occasionally | 🞎 |  |  |  |
| Rarely | 🞎 |  |  |  |
| Never | 🞎 |  |  |  |
| **Whilst in the sun, did you use suntan lotion/sunscreen?** | | | | |
| Yes | 🞎 |  |  |  |
| No | 🞎 |  |  |  |
| Sometimes | 🞎 |  |  |  |
| If yes or sometimes, name the lotion used most often: | | |  |  |
|  |  | SPF: |  |  |
| Other lotions and SPFs used: |  |  |  |  |
|  |  |  |  |  |
|  |  |  |  |  |
| **During the past two years, have you had sunburn at all?** | | | |  |
| Yes | 🞎 |  |  |  |
| No | 🞎 |  |  |  |
|  |  |  |  |  |
| **Have you ever used a sun-bed?** |  |  |  |  |
| Yes | 🞎 |  |  |  |
| No | 🞎 |  |  |  |
| **If yes**, how often: ……………….. times a week for ……………….. months | | | | |
| **Have you ever used a UV sunlamp?** | |  |  |  |
| Yes | 🞎 |  |  |  |
| No | 🞎 |  |  |  |
| **If yes**, how often: ……………….. times a week for ……………….. months | | | | |


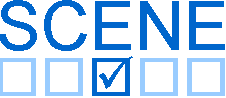


*We would also like to ask you a few questions about your background*

| **1. Are you male or female?** |  |  | **5. Do you have any children?** |  |
| --- | --- | --- | --- | --- |
| (a) Male | 🞎 |  | (a) No | 🞎 |
| (b) Female | 🞎 |  | (b) Yes | 🞎 |
| **2. How old are you?** |  |  | **6. What is the highest level of education you have completed?** |  |
| (a) less than 18 | 🞎 |  | (a) primary school | 🞎 |
| (b) 18-24 | 🞎 |  | (b) secondary school | 🞎 |
| (c) 25-34 | 🞎 |  | (c) O level/GCSEs | 🞎 |
| (d) 35-44 | 🞎 |  | (d) A levels | 🞎 |
| (e) 45-54 | 🞎 |  | (e) Technical or trade certificate | 🞎 |
| (f) 55-64 | 🞎 |  | (f) Diploma | 🞎 |
| (g) 65-74 | 🞎 |  | (g) Degree | 🞎 |
| (h) more than 75 | 🞎 |  | (h) Post-graduate degree | 🞎 |
| **3. Are you:** |  |  | **7. What is your job?** *If you are not working now, what was your usual job? (please be specific)* | |
| (a) single | 🞎 |  |
| (b) married | 🞎 |  |
| (c) living as married | 🞎 |  |  |  |
| (d) separated | 🞎 |  |  |  |
| (e) divorced | 🞎 |  |  |  |
| (f) widowed | 🞎 |  | **8. If you have a partner, what is his/her job?** *If he/she is not working now, what was his/her usual job? (please be specific)* | |
| **4. What is your ethnic origin?** |  |  |
| (a) White | 🞎 |  |  |  |
| (b) Black Caribbean | 🞎 |  |  |  |
| (c) Black African | 🞎 |  |  |  |
| (d) Black other | 🞎 |  |  |  |
| (e) Indian | 🞎 |  | **9. Are you currently:** |  |
| (f) Pakistani | 🞎 |  | (a) employed full time | 🞎 |
| (g) Bangladeshi | 🞎 |  | (b) employed part time | 🞎 |
| (h) Chinese | 🞎 |  | (c) unemployed | 🞎 |
| (i) Asian - other | 🞎 |  | (d) full time homemaker | 🞎 |
| Please specify: |  |  | (e) retired | 🞎 |
| (j) Any other ethnic group | 🞎 |  | (f) student | 🞎 |
| Please specify: |  |  | (g) disabled or too ill to work | 🞎 |


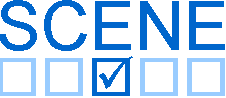


|  | **Please use the section below to tell us about your recent experiences of skin cancer**  **care and any issues or support needs that you have identified:** | | | | | |  |
| --- | --- | --- | --- | --- | --- | --- | --- |
|  |  |  |  |  |  |  |  |
|  |  |  |  |  |  |  |  |
|  |  |  |  |  |  |  |  |
|  |  |  |  |  |  |  |  |
|  |  |  |  |  |  |  |  |
|  |  |  |  |  |  |  |  |
|  |  |  |  |  |  |  |  |
|  |  |  |  |  |  |  |  |
|  |  |  |  |  |  |  |  |
|  |  |  |  |  |  |  |  |
|  |  |  |  |  |  |  |  |

If you would like to receive a summary of the findings of this study, please tick this box 🞎 and provide us with a name and address to which we can post a leaflet:

…………………………………………………………………………………………………………………………………………………………………...

…………………………………………………………………………………………………………………………………………………………………….

…………………………………………………………………………………………………………………………………………………………………….

**Thank you very much for taking the time to complete this questionnaire,**

**now please return to the researcher/in the prepaid envelope provided.**
